# The role of prophylactic use of low molecular weight heparin or aspirin in thromboembolic events in primary membranous nephropathy

**DOI:** 10.1080/0886022X.2019.1635030

**Published:** 2019-07-04

**Authors:** Peimei Zou, Hang Li, Jianfang Cai, Chao Li, Zhenjie Chen, Xuewang Li

**Affiliations:** a Blood Purification Center, Beijing ChaoYang Hospital, Capital Medical University, Beijing, China;; b Department of Nephrology, Peking Union Medical College Hospital, Chinese Academy of Medical Sciences and Peking Union Medical College, Beijing, China;; c Department of Nephrology, Dongzhimen Hospital, Beijing University of Chinese Medicine, Beijing, China

**Keywords:** Primary membranous nephropathy, low molecular weight heparin, aspirin, thromboembolism, arterial

## Abstract

**Objective:** The aim of this study is to investigate the role of prophylactic anticoagulation regimens based on low molecular weight heparin (LMWH) or aspirin in thromboembolic events in patients with primary membranous nephropathy (PMN).

**Methods:** A total of 717 patients with PMN were consecutively enrolled in this retrospective study. The propensity score matching method was utilized to adjust for the selection bias inherent in an analysis of outcomes, which was stratified by the anticoagulation prophylaxis regimen.

**Results:** According to the anticoagulation prophylaxis regimen, patients were assigned into three groups: only LMWH therapy (L + A−, *n* = 53), only aspirin therapy (L − A+, *n* = 97), and no therapy of LMWH or aspirin (L − A−, *n* = 567). After performing 1:1 match, 37 patients were selected in the L + A − group and the L − A− group, respectively, and 94 patients were selected in the L − A+ group and the L − A− group, respectively. It showed that the prophylactic use of LMWH had no protective effects on arterial thromboembolic events (ATEs) (10.8% vs. 21.6%, *p* = .21) or venous thromboembolic events (VTEs) (8.1% vs. 10.8%, *p* = .69). The incidence of VTEs in the L − A+ group was lower than the L − A− group (2.1% vs. 10.6%, *p* = .02), while there were no significant differences in the incidences of ATEs between the L − A+ group and the L − A− group (5.3% vs. 7.4%, *p* = .55).

**Conclusions:** The prophylactic use of LMWH showed no benefits on the incidence of ATEs or VTEs in patients with PMN. Aspirin effectively decreased the incidence of VTEs, without effects on the occurrence of ATEs.

## Introduction

Primary membranous nephropathy (PMN) is associated with high risks of thromboembolism events, both arterial thromboembolic events (ATEs) and venous thromboembolic events (VTEs) [1–4]. ATEs mainly consist of acute myocardial ischemic events and infarction, and thrombotic ischemic stroke (IS). The thrombophilic state with nephrotic syndrome and classic risk factors for atherosclerosis were associated with the onset of ATEs, while hypoalbuminemia is the most important independent risk factor of VTEs [4,5]. These serious complications significantly increased the morbidity and mortality of patients with PMN. Despite the well-established risks of VTEs in PMN, the most effective method of VTE prophylaxis is still unclear [6]. High incidence of ATEs in PMN attracted our attention recently; however, the preventative method of ATEs was limited.

Therefore, the aim of this study was to investigate the role of prophylactic anticoagulation regimens based on low molecular weight heparin (LMWH) or aspirin in thromboembolism events in patients with PMN. To our knowledge, this is the first study about anticoagulation prophylaxis in PMN.

## Materials and methods

We retrospectively enrolled patients pathologically diagnosed as PMN by renal biopsy from January 2004 to June 2016 at the Peking Union Medical College Hospital. The exclusion criteria were (1) patients with malignant tumors, autoimmune diseases, serious mental diseases and hematological diseases; (2) patients with comorbidity of other pathological types of glomerular diseases; (3) patients who presented with arterial or venous thrombotic events at the time of diagnosis of PMN. This study was approved by the ethics committee of the Peking Union Medical College Hospital (No. S-K120).

Clinical characteristics at the time of biopsy were collected, including gender, age, duration of the disease, history of smoking, history of diabetes, hypertension, and previous onset of ATEs, proteinuria (24 h-UP), serum albumin, serum creatinine, total cholesterol, triglycerides, and low density lipoprotein-cholesterol (LDL-C). The estimated glomerular filtration rate (eGFR) was calculated using the Chronic Kidney Disease Epidemiology Collaboration (CKD-EPI) formula [7]. Prophylactic anticoagulation regimens of patients were recorded. In addition, we also collected treatments for PMN, including blood pressure control, administration of angiotensin converting enzyme inhibitors (ACEIs) or angiotensin receptor blockers (ARBs), glucocorticoids and immunosuppressive agents. Nephrotic syndrome (NS) was identified as proteinuria ≥3.5 g/24 h and hypoalbuminemia (≤30 g/L) [8]. Medical records were reviewed to collect thromboembolic events during prophylactic anticoagulation treatment. VTEs were consisted of deep vein thrombosis (DVT), renal vein thrombosis (RVT), and pulmonary embolism (PE), and ATEs included coronary heart disease (CHD), IS and peripheral artery disease (PAD). The diagnoses of DVT, RVT and PAD were confirmed by compression sonography and color-Doppler ultrasound. The diagnosis of PE was performed by CT pulmonary angiography. CHD with any of myocardial infarction, angina pectoris or silent myocardial ischemia was confirmed by physicians, based on the clinical manifestations and supportive electrocardiogram and cardiac enzyme data. IS was diagnosed by medical imaging of CT or MRI.

Statistical analysis was performed by SPSS (version 22, IBM, Armonk, NY). Continuous variables were presented as mean ± SD or median with interquartile ranges (IQR), and the differences were evaluated by the Student *t*-test, the Mann–Whitney *U* test, the Kruskal–Wallis test, or univariate ANOVA, depending on the normality of the data and levels of the outcome variable. Categorical variables were presented with percentages, and the differences were compared using chi-square test or Fisher’s test. Propensity score matching method was utilized to adjust for the selection bias inherent in an analysis of outcomes, which was stratified by the anticoagulation prophylaxis regimen. The following variables were selected to calculate the propensity score: age, sex, history of smoking, history of diabetes, hypertension, and previous onset of ATEs, proteinuria, serum albumin, cholesterol, and low density lipoprotein–cholesterol (LDL-C). In our propensity analyses, a 1:1 matching ratio was used. Subsequently, covariate balance between the matched groups was examined.

## Results

A total of 749 patients were pathologically diagnosed as PMN by renal biopsy from January 2004 to June 2016 at Peking Union Medical College Hospital. Thirty-two patients who presented with an arterial or venous thrombotic event at the time of diagnosis of PMN were excluded. Finally, 717 patients (58% were male) were enrolled in the study. The mean age was 47.4 ± 14.8 years. Median observation period was 39.5 (24.5, 61.5) months. 84 patients (11.7%) experienced thromboembolic events during the observation period, of whom 47 had ATEs and 37 had VTEs. ATEs were consisted of CHD (*n* = 26, 55%) and IS (*n* = 21, 45%).

According to the anticoagulation prophylaxis regimen, patients were divided into three groups: only LMWH therapy (L + A−, *n* = 53), only aspirin therapy (L − A+, *n* = 97), and no therapy of LMWH or aspirin (L − A−, *n* = 567). Patients in the L + A − group were received LMWH 4000–6000 IU by subcutaneous injections, and then switched to warfarin therapy or not. For patients who were switched to warfarin, aiming to an international normalized ratio (INR) of 1.8–2.5. Patients in L − A+ group were received aspirin treatment with 75 mg or 100 mg daily. Baseline data showed that serum albumin level was the highest in the L − A− group (27.8 ± 6.5 g/L), while patients who had ever been received therapy of LMWH (L + A − group) had the relative lower serum albumin levels (22.0 ± 6.6 g/L) (Table 1).

**Table 1.** Baseline characteristics of patients in each group.

	L + A− (*n* = 53)	L − A+ (*n* = 97)	L − A− (*n* = 567)	*p* Value
Male, *n* (%)	41 (77)	60 (62)	315 (56)	.01
Age, years	48.4 ± 14.8	52.6 ± 13.3	46.4 ± 14.8	.34
History of				
Smoking, *n* (%)	23 (43)	34 (35)	157 (28)	.03
Diabetes, *n* (%)	6 (11)	18 (19)	61 (11)	.09
Hypertension, *n* (%)	25 (47)	73 (75)	268 (47)	<.001
Prior ATE, *n* (%)	3 (6)	16 (16)	15 (3)	<.001
Observation time, m	49.0 (28.1, 80.0)	38.1 (29.7, 65.2)	39.5 (23.9, 59.3)	.04
Proteinuria, g/d	7.21 (3.83, 12.11)	5.25 (3.28, 7.60)	5.10 (3.05, 8.50)	<.001
Serum albumin, g/l	22.0 ± 6.6	27.2 ± 5.8	28.1 ± 6.5	<.001
eGFR, ml/min/1.73 m^2^	92.35 ± 20.63	92.83 ± 20.22	98.51 ± 22.15	.05
Total cholesterol, mmol/l	9.15 ± 2.92	8.00 ± 2.22	7.85 ± 2.38	.01
Triglycerides, mmol/l	2.49 (1.84, 3.02)	2.63 (1.80, 3.83)	2.31 (1.63, 3.37)	.44
LDL-C, mmol/l	4.76 (3.80, 7.66)	4.79 (3.60, 5.96)	4.64 (3.55, 6.03)	.11
ATEs, *n* (%)	2 (3.8)	8 (8.2)	35 (6.2)	.55
VTEs, *n* (%)	7 (13.2)	2 (2.1)	27 (4.8)	.01

L: low molecular weight heparin; A: aspirin; eGFR: estimated glomerular filtration rate; LDL-C: low-density lipoprotein cholesterol; ATEs: arterial thromboembolic events; VTEs: venous thromboembolic events.

After performing 1:1 match, 37 patients were selected in the L + A − group and the L − A− group, respectively. There were no statistical differences in variables between the two groups, including sex, history of smoking, proteinuria, serum albumin and cholesterol, indicating a high degree of homogeneity in the distribution of both groups. Results showed that the prophylaxis use of LMWH had no protective effects on thromboembolic events (10.8% vs. 21.6%, *p* = .21; 8.1% vs. 10.8%, *p* = .69) (Table 2).

**Table 2.** Comparison of L + A − group and L − A− group, and after propensity score matching.

	Full data set			Propensity score matched patients		
	L + A− (*i* = 53)	L − A− (*n* = 567)	*p* Value	L + A− (*n* = 37)	L − A− (*n* = 37)	*p* Value
Male, *n* (%)	41 (77)	315 (56)	.002	29 (78)	23 (62)	.13
Age, years	48.4 ± 14.8	46.4 ± 14.8	.34	49.2 ± 14.9	47.9 ± 14.9	.72
History of						
Smoking, *n* (%)	23 (43)	157 (28)	.02	14 (38)	15 (41)	.81
Diabetes, *n* (%)	6 (11)	61 (11)	.90	6 (16)	2 (5)	.13
Hypertension, *n* (%)	25 (47)	268 (47)	1.00	18 (49)	18 (49)	1.00
Prior ATE, *n* (%)	3 (6)	15 (3)	.21	3 (8)	2 (5)	.64
Proteinuria, g/d	7.21 (3.83, 12.11)	5.10 (3.05, 8.50)	.01	6.62 (3.99, 11.63)	5.85 (3.56, 9.68)	.37
Serum albumin, g/l	22.0 ± 6.6	28.1 ± 6.5	<.001	22.4 ± 6.4	25.3 ± 7.7	.09
eGFR, ml/min/1.73 m^2^	92.35 ± 20.63	98.51 ± 22.15	.05	92.71 ± 18.85	95.41 ± 21.95	.57
Total cholesterol, mmol/l	9.15 ± 2.92	7.85 ± 2.38	.01	9.05 ± 2.95	8.54 ± 3.83	.53
Triglycerides, mmol/l	2.49 (1.84, 3.02)	2.31 (1.63, 3.37)	.50	2.49 (1.86, 3.05)	2.37 (1.60, 3.21)	.60
LDL-C, mmol/l	4.76 (3.80, 7.66)	4.64 (3.55, 6.03)	.19	4.64 (3.80, 7.34)	4.47 (3.43, 6.37)	.51
ATEs, *n* (%)	2 (3.8)	35 (6.2)	.48	4 (10.8)	8 (21.6)	.21
VTEs, *n* (%)	7 (13.2)	27 (4.8)	.01	3 (8.1)	4 (10.8)	.69

L: low molecular weight heparin; A: aspirin; eGFR: estimated glomerular filtration rate; LDL-C: low-density lipoprotein cholesterol; ATEs: arterial thromboembolic events; VTEs: venous thromboembolic events.

For propensity score matching, variables including age, eGFR, history of diabetes, hypertension, and previous onset of ATEs were matched. 94 patients were selected in the two groups, respectively. As shown in Table 3, the incidence of VTEs in the L − A+ group was lower than the L − A− group (2.1% vs. 10.6%, *p* = .02), while incidences of ATEs had no significant differences between the two groups (5.3% vs. 7.4%, *p* = .55) (Table 3).

**Table 3.** Comparison of the L − A+ group and the L − A− group, and after propensity score matching.

	Full data set			Propensity score matched patients		
	L − A+ (*n* = 97)	L − A− (*n* = 567)	*p* Value	L − A+ (*n* = 94)	L − A− (*n* = 94)	*p* Value
Male, *n* (%)	60 (62)	315 (56)	.24	58 (62)	52 (55)	.37
Age, years	52.6 ± 13.3	46.4 ± 14.8	<.001	52.3 ± 13.4	48.1 ± 16.0	.05
History of						
Smoking, *n* (%)	34 (35)	157 (28)	.14	33 (35)	29 (31)	.54
Diabetes, *n* (%)	18 (19)	61 (11)	.03	18 (19)	15 (16)	.57
Hypertension, *n* (%)	73 (75)	268 (47)	<.001	61 (65)	49 (52)	.08
Prior ATE, *n* (%)	16 (16)	15 (3)	<.001	15 (16)	15 (16)	1.00
Proteinuria, g/d	5.25 (3.28, 7.60)	5.10 (3.05, 8.50)	.36	5.70 (3.76, 7.79)	6.12 (3.75, 9.37)	.35
Serum albumin, g/l	27.2 ± 5.8	28.1 ± 6.5	.22	27.1 ± 5.8	27.5 ± 7.3	.68
eGFR, ml/min/1.73 m^2^	92.83 ± 20.22	98.51 ± 22.15	.02	93.38 ± 19.57	98.29 ± 23.13	.12
Total cholesterol, mmol/l	8.00 ± 2.22	7.85 ± 2.38	.63	8.03 ± 2.23	8.21 ± 2.58	.66
Triglycerides, mmol/l	2.63 (1.80, 3.83)	2.31 (1.63, 3.37)	.07	2.67 (1.81, 3.93)	2.46 (1.73, 3.55)	.51
LDL-C, mmol/l	4.79 (3.60, 5.96)	4.64 (3.55, 6.03)	.92	4.92 (3.56, 6.00)	4.97 (3.82, 5.83)	.75
ATEs, *n* (%)	8 (8.2)	35 (6.2)	.44	5 (5.3)	7 (7.4)	.55
VTEs, *n* (%)	2 (2.1)	27 (4.8)	.23	2 (2.1)	10 (10.6)	.02

L: low molecular weight heparin; A: aspirin; eGFR: estimated glomerular filtration rate; LDL-C: low-density lipoprotein cholesterol; ATEs: arterial thromboembolic events; VTEs: venous thromboembolic events.

## Discussion

Studies about prophylactic anticoagulation regimens with LMWH or aspirin in thromboembolism events were mainly in patients with tumors, surgical patients, and hospitalized internal patients [9–12]. In nephrology, the role of prophylactic anticoagulants in the treatment of patients with nephrotic syndrome is a prime example of the conundrum of uncertainty [6]. However, the related studies in patients with PMN are limited. To our knowledge, this is the first study about the application of LMWH or aspirin in patients with PMN.

Our previous study [4] with the same cohort indicated that severe proteinuria, aging, smoking, hypertension and prior ATE in patients with PMN were associated with the incidence of ATEs. Aging was noted as the independent risk factor for ATEs (*p* < .001), and hypoalbuminemia was the dominant independent risk factor for VTEs (*p* < .03). The propensity matching analyses in our present study showed that the prophylactic use of LMWH had no benefits on thromboembolic events in patients aged 49.2 ± 14.9 years and with the serum albumin level of 22.4 ± 6.4 g/L; in patients aged 52.3 ± 13.4 years and with the serum albumin level of 27.1 ± 5.8 g/L, the prophylactic use of aspirin decreased the incidence of VTEs, but had no effect on the incidence of ATEs.

Thromboembolism is one of the common complications of NS, especially in membranous nephropathy. However, the appropriate prophylactic anticoagulation treatment is still controversial [13]. The 2012 KDIGO (Kidney Disease: Improving Global Outcomes) guideline suggested that patients with IMN and NS, with marked reduction in serum albumin (<25 g/l) and additional risks for thrombosis (e.g., congestive heart failure; prolonged immobilization; morbid obesity; abdominal, orthopedic, or gynecologic surgery), were considered for prophylactic anticoagulant therapy, using oral warfarin (Grade 2 C) [14]. Short-term full dose of heparin was used to prolong the coagulation time before the treatment of oral warfarin. However, the detail dosage and the course were ambiguous. Dr Glassock considered that the INR during maintenance therapy should be targeted to 1.8–2.0, and the treatment should be continued as long as the patient is nephrotic (unless, of course, a serious hemorrhagic event ensues) [6].

There was a research constructing a Markov decision model from an inception cohort of 898 patients with PMN, evaluating the potential benefits of prophylactic anticoagulation (venous thromboembolic events prevented) relative to the risks (major bleeds), and then providing an approach to the decision of prophylactic anticoagulation personalized to the individual’s needs [15]. Benefit-to-risk ratios were predicted according to bleeding risk and serum albumin. Finally, it showed that the ratio increased with worsening hypoalbuminemia from 4.5:1 for an albumin under 3 g/dl to 13.1:1 for an albumin under 2 g/dl in patients with low bleeding risk. However, this tool was only applicable to PMN with nephrotic syndrome treated with prolonged oral warfarin (to a target INR of 2–3), and the overall effectiveness of the computerized algorithms in preventing morbidity and mortality had not been prospectively evaluated. It also ignored the additional risks for thrombosis, such as prolonged immobilization, morbid obesity, congestive heart failure, and abdominal surgery [16]. A retrospective study of 143 patients presenting with nephrotic syndrome secondary to biopsy-proven PMN, MCD, and FSGS demonstrated that, the regimen of prophylactic antiplatelet or anticoagulant therapy appears effective in preventing VTE in nephrotic syndrome, with relatively few hemorrhagic complications [17]. They designed anticoagulation prophylaxis regimens for patients according to the serum albumin: patients presenting with serum albumin levels <2.0 g/dl received LMWH by subcutaneous injections (enoxaparin, 20 mg once daily or equivalent formulation); patients with hypoalbuminemia <2.0 g/dl for >3 months were considered for a switch to low-dose warfarin, targeting to an INR of 1.5–2.5; patients in whom albumin level improved to 2.0–3.0 g/dl were switched to aspirin with 75 mg daily. When the serum albumin was >3.0 g/dl, the prophylaxis regimen was terminated.

Our study showed that, in patients aged 49.2 ± 14.9 years and presented with the serum albumin level of 22.4 ± 6.4 g/L, the prophylactic use of LMWH had no benefits on the ATEs or VTEs. This conclusion was not accordant with previous studies, which may be related to the following limitations: (1) patients with asymptomatic VTEs without routinely follow up, and patients with mild symptoms of ATEs were ignored; (2) the relative small sample size. After performing 1:1 matching, there were only 37 patients in L + A − group and L − A− group, respectively. (3) Variable usage of LMWH and warfarin, including dosage, course, and the maintenance.

The antiplatelet agent aspirin usage is associated with an increased risk of gastrointestinal bleeding [18]. Guideline recommendations of aspirin use as primary prevention of cardiovascular diseases were based on the net benefits (the balance of beneficial effects and bleeding hazards). In the primary prevention trials, aspirin allocation yielded a 12% proportional reduction in serious vascular events, mainly due to nearly a fifth of the reduction in non-fatal myocardial infarction; while the net effect on stroke was not significant [19]. Multiple meta-analysis studies reflect risks but no benefits of aspirin for the prevention of a first stroke in the general population [19–21]. There is no evidence that antiplatelet medications reduce the risks of stroke in the general population. The most recent 2014 AHA/ASS (the American Heart Association/American Stroke Association) guideline for the primary prevention of cardiovascular diseases also recommends aspirin for primary cardiovascular prevention (including but not specific to stroke) in those with a 10-year risk >10%. A cardiovascular risk calculator to assist in estimating 10-year risk can be found online at http://my.americanheart.org/cvriskcalculator (including age, sex, race, total cholesterol, HDL cholesterol, systolic blood pressure, blood pressure lowering medication use, diabetes status, and smoking status) (Class IIa; Level of Evidence A) [18].

Patients with PMN are also at high risk of cardiovascular events [3–5]. We firstly tried to explore aspirin in the primary prevention of cardiovascular diseases in patients with PMN. Our study indicated that the prophylactic use of aspirin had no effects on the incidence of ATEs. The negative result may due to characteristics of this study below: First, retrospective design may result the ignorance of mild symptoms of ATEs. Secondary, according to the cardiovascular risks calculator to assist in estimating 10-year risk, patients with PMN were mostly with a 10-year risk >10%; however, in ATEs occurred in PMN, IS accounted for 45%, while the net effects in the primary prevention of aspirin on stroke was not significant. Lastly, the aspirin was not strictly long-term administrated.

In nephrotic syndrome, platelet hyperaggregability and platelet dysfunction were observed [1,22]. Clinical evidence showed that platelets contributed to the initiation and progression of venous thrombosis and aspirin inhibited thrombin formation and thrombin-mediated coagulant reactions [23–27]. Aspirin has been shown significant benefits in preventing VTE in high-risk medical patients [9,28,29]. The recent 2012 ACCP (the American College of Chest Physicians) recommendations for surgical patients encouraged the use of aspirin as an alternative to LMWH [30,31]. Our study demonstrated that aspirin could effectively decrease incidence of VTEs in patients with PMN, which was consistent with results of one previous study [17]. However, it is not a priority selection to use aspirin as the primary prevention of venous thrombosis. The current approach is to recommend aspirin on patients who cannot receive other antithrombotic agents [32]. Our study also provided the supported clinical evidence.

However, there are several limitations in our study. It is a retrospective study, and the data of hemorrhagic complications is lacked. The anticoagulation prophylaxis regimens were not rigorously administrated. In summary, this study showed that, the prophylactic use of aspirin decreased the incidence of VTEs, but had no effects on the incidence of ATEs; LMWH had no benefits on the thromboembolic events. It provides valuable clinical evidence through a single center’s experience, but the limitations should also be recognized. Prophylactic anticoagulant therapy is still a clinical conundrum in nephrotic syndrome, and well-designed multi-center prospective studies were further needed.
